# The risk of gastric cancer according to changes in smoking status among Korean men

**DOI:** 10.4178/epih.e2022086

**Published:** 2022-10-07

**Authors:** Sung Keun Park, Min-Ho Kim, Chang-Mo Oh, Eunhee Ha, Eun Hye Yang, Woo Yeon Hwang, Ann Hee You, Jae-Hong Ryoo

**Affiliations:** 1 Center for Cohort Studies, Total Healthcare Center, Kangbuk Samsung Hospital, Sungkyunkwan University School of Medicine, Seoul, Korea; 2 Informatization Department, Ewha Womans University Seoul Hospital, Seoul, Korea; 3 Departments of Preventive Medicine, Kyung Hee University School of Medicine, Seoul, Korea; 4 Department of Occupational and Environment Medicine, Ewha Womans University College of Medicine, Seoul, Korea; 5 Department of Occupational and Environmental Medicine, Kyung Hee University Hospital, Seoul, Korea; 6 Department of Obstetrics and Gynecology, Kyung Hee University Hospital, Seoul, Korea; 7 Department of Anesthesiology and Pain Medicine, Kyung Hee University Hospital, Seoul, Korea; 8 Departments of Occupational and Environmental Medicine, Kyung Hee University School of Medicine, Seoul, Korea

**Keywords:** Smoking, Gastric cancer, Current smoker, Former smoker, Pack year

## Abstract

**OBJECTIVES:**

Smoking is a risk factor for gastric cancer. Studies have shown that the risk of gastric cancer can vary by smoking status and smoking amount at a single point in time. However, few data have been reported about the effect of changes in smoking status over time on the risk of gastric cancer.

**METHODS:**

This study collected data from the National Health Insurance Corporation in Korea on 97,700 Korean men without gastric cancer who underwent health check-ups from 2002 to 2013. The smoking status (never smoked, quit smoking, and currently smoking) of study participants was assessed in 2003-2004 and 2009, and the results were categorized into 7 groups: never-never, never-quit, never-current, quit-quit, quit-current, current-quit, and current-current. Participants were followed until 2013 to identify incident gastric cancer. A multivariate Cox proportional hazard model was used to calculate adjusted hazard ratios (HRs) and 95% confidence intervals (CIs) for incident gastric cancer according to changes in smoking status and smoking amount (pack-years).

**RESULTS:**

Compared with group 1 (never-never), participants currently smoking in 2009 (never-current, quit-current, and current-current) had higher HRs for gastric cancer (never-quit: 1.077; 95% CI, 0.887 to 1.306, never-current: 1.347; 95% CI, 0.983 to1.846, quit-quit: 1.086; 95% CI, 0.863 to 1.366, quit-current: 1.538; 95% CI, 1.042 to 2.269, current-quit: 1.339; 95% CI, 1.077 to 1.666, and current-current: 1.589; 95% CI, 1.355 to 1.864, respectively). The risk for gastric cancer was highest in heavy smokers, followed by moderate smokers.

**CONCLUSIONS:**

In all categories of smoking status, current smoking was associated with the highest risk of gastric cancer. Heavy smoking was associated with an increased risk of gastric cancer, even in former smokers.

## GRAPHICAL ABSTRACT

[Fig f1-epih-44-e2022086]

## INTRODUCTION

Although the incidence and mortality of gastric cancer have gradually decreased worldwide, gastric cancer is still the fifth most common malignancy and third leading cause of cancer death globally [1]. East Asia, including Korea, is characterized by a high prevalence of gastric cancer, accounting for three-quarters of the global incident cases [2]. Thus, there is interest in identifying potentially modifiable environmental factors and determining their role in the development of gastric cancer.

Smoking is a major concern in global public health. Despite a global decline in the proportion of smokers, the total number of smokers has increased due to population growth [3]. Smoking is known to contribute to the pathogenesis of diverse cancers and is a key risk factor for gastric cancer. Epidemiological studies have consistently reported that current smoking is associated with a 1.5 to 2.5-fold increase in the risk of gastric cancer compared with never having smoked [4-6]. Although the intensity and duration of smoking have been associated with the risk of gastric cancer in a dose-response pattern [7-9], this association has not been confirmed in former smokers. A study of East Asians showed that former smokers had a risk of gastric cancer like that of those who have never smoked [6]. Another study indicated that the risk of gastric cancer decreased with an increase in the duration of smoking cessation and approached the level of never smokers after 10 years [10]. Although smoking is a known risk factor, these studies suggest that the risk of gastric cancer can vary by smoking status [6-10].

Most studies have investigated the association between smoking and gastric cancer based on smoking status or smoking amount at a single point in time. However, smoking status can change over time. Social policies and anti-smoking campaigns recognize the common cycles of smoking status (e.g., cessation, a return to smoking, and repeat cessation) that result in smoking status changes. However, no studies have yet assessed the risk of gastric cancer according to these changes in smoking status.

The purpose of our study was to identify the effect of changes in smoking status on the risk of gastric cancer. Therefore, we investigated the risk of incident gastric cancer according to changes in 3 categories of smoking status (never smoked, quit smoking, and currently smoking) over time.

## MATERIALS AND METHODS

### Data sources

In Korea, the National Health Insurance Corporation (NHIC) provides the National Health Insurance Service (NHIS), which covers 97% of the Korean population. Medical information from the NHIS, which represents the medical service usage of the entire Korean population, is stored in the National Health Information Database (NHID) [11], including data from the annual or biennial national health check-ups of all adults aged > 40 years.

Most Korean medical institutions have a contract with the NHIC to provide the medical information of their healthcare users and patients. Therefore, the NHID contains the medical information and socio-demographic variables of the Korean population collected from health care utilization and health check-ups. In recent years, the NHIS in Korea has provided access to the sampled database for research purposes after deleting personal identification information. The sampled data includes information from health check-ups and is linked with Statistics Korea, which contains data on the development of gastric cancer in Korea.

### Study participants

Based on the NHID information, 194,444 subjects who underwent health check-ups between 2003-2004 and in 2009 and had valid cigarette smoking records were included in our study. Of these, 10,742 participants whose smoking status was not valid (quit-never and current-never) were excluded. In addition, we excluded 2,347 participants who were diagnosed with gastric cancer between 2002 and the date before their medical health examination in 2009, as well as 83,655 women participants. The final analysis included 97,700 participants who were monitored for incident gastric cancer. The total follow-up period was 423,288.7 person-years and the average follow-up period was 4.33 (standard deviation [SD], 0.53) person-years.

### Definition of changes in smoking status and smoking amount

Changes in smoking status were assessed by answers to a selfadministered questionnaire during the biennial health check-up. According to responses about smoking status at the time of their health check-up in 2003-2004 and again in 2009, study participants were categorized into 3 groups: never (participants who never smoked), quit (participants who had quit smoking) and current (participants currently smoking). Study participants were then further categorized into 7 groups based on their change in smoking status between the first examination (2003-2004) and the second examination (2009) as follows: group 1 (never-never), group 2 (never-quit), group 3 (never-current), group 4 (quit-quit), group 5 (quit-current), group 6 (current-quit), and group 7 (current-current). Smoking amount was determined in 2009 and was defined in pack-years (1 pack year= 1 year× 1 pack per day). Participants were divided by smoking amount into 4 groups: never smokers, light smokers (0-10 pack-years), moderate smokers (10-20 pack-years), and heavy smokers (> 20 pack-years).

### Health survey examinations and laboratory measurements

The NHIC general health examination is conducted in 2 stages. The first stage is a comprehensive screening test to determine the presence or absence of disease among the general population without symptoms. The second stage includes consultation screening tests and a more detailed examination to confirm the presence of disease. These health examinations also include a questionnaire covering lifestyle and past medical history. Our study data included information provided by these questionnaires, as well as anthropometric measurements and laboratory measurements. Alcohol intake was defined as consuming alcohol > 3 times per week. Physical activity was defined as moderate-intensity physical activity (at least 30 minutes per day > 4 days each week) or vigorousintensity physical activity (at least 20 minutes per day > 4 days each week). Body mass index (BMI) was calculated as weight (kg) divided by height squared (meters).

Systolic blood pressure (BP) and diastolic BP were measured by trained examiners. The following laboratory data were measured when these participants underwent health examinations: fasting blood glucose, total cholesterol, triglyceride, high-density lipoprotein cholesterol, low-density lipoprotein cholesterol, aspartate aminotransferase, alanine aminotransferase and gamma-glutamyl transferase (GGT) levels.

### Outcome definitions

The NHID was linked to data on diagnosed diseases from Statistics Korea. In this study, the entry date was the time of the first health check-up after 2009, and the last follow-up date for diagnosis of gastric cancer was December 31, 2013. The primary endpoint of this study was the identification of gastric cancer based on the International Classification of Diseases, 10th revision, Clinical Modification (ICD-10-CM) codes registered in the NHID. Gastric cancer was diagnosed based on biopsy results after endoscopy with an ICD-10 code assigned at the time (C16).

### Statistical analysis

Data were expressed as mean±SD or median (interquartile range) for continuous variables and percentages for categorical variables. One-way analysis of variance and the chi-square test were used to analyze statistical differences among the characteristics of the study participants at the time of enrollment in relation to changes in smoking status. Person years were calculated as the sum of the follow-up years for all participants from the baseline entry date until the date of gastric cancer diagnosis or until December 31, 2013, whichever came first.

To evaluate associations among the 7 groups according to changes in smoking status and incident gastric cancer, we used Cox proportional hazards models to estimate adjusted hazard ratios (HRs) and 95% confidence intervals (CIs) for incident gastric cancer. The Cox proportional hazards models were adjusted for multiple confounding factors. In the multivariate-adjusted models, we included variables that might confound the relationship between smoking status change and smoking amount and incident gastric cancer, such as age, BMI, systolic BP, fasting blood glucose level, total cholesterol level, alcohol intake, and physical activity. To test the validity of the Cox proportional hazard models, we checked the proportional hazard assumption. The proportional hazard assumption was assessed using a log-minus-log survival function and was found to be graphically unviolated. A p-value < 0.05 indicated statistical significance. All statistical analyses were performed using SAS version 9.4 (SAS Institute Inc., Cary, NC, USA).

### Ethics statement

Ethics approval for the study protocol and analysis of the data was obtained from the Institutional Review Board (IRB) of Kyung Hee University Hospital. The informed consent requirement was exempted by the IRB because researchers retrospectively accessed an anonymized database for analysis purposes.

## RESULTS

During 423,288.7 person-years of follow-up, 1,119 (1.15%) incident cases of gastric cancer developed between 2009 and 2013. The baseline characteristics of the study participants in relation to the 7 groups of smoking status change are presented in Table 1. At baseline, the mean± SD age and BMI of study participants were 57.2± 8.5 years and 24.0± 2.7 kg/m2, respectively. Group 7 (current-current smoking) was characterized by more alcohol intake, higher triglyceride levels, higher GGT levels, a higher proportion of heavy smokers, lower physical activity, and a higher incidence of gastric cancer than other groups. In terms of other variables, despite statistically significant differences among groups, we did not find noteworthy differences or specific directions of relationships among groups.

Table 2 shows the HRs and 95% CIs for the incidence of gastric cancer according to the 7 groups classified by changes in smoking status. In the unadjusted model, group 7 (current-current) had a higher HR (95% CI) for incident gastric cancer than group 1 (never-never): group 1 (reference); group 2, 1.078 (95% CI, 0.891 to 1.305); group 3, 1.153 (95% CI, 0.853 to 1.558); group 4, 0.891 (95% CI, 0.711 to 1.116); group 5, 1.179 (95% CI, 0.806 to 1.725); group 6, 1.056 (95% CI, I0.853 to 1.308); and group 7, 1.217 (95% CI, 1.045 to 1.416). The multivariable-adjusted model showed that groups 5-7 were significantly associated with incident gastric cancer, when compared with group 1: never-quit, 1.077 (95% CI, 0.887 to 1.306), never-current, 1.347 (95% CI, 0.983 to 1.846), quit-quit, 1.086 (95% CI, 0.863 to 1.366), quit-current, 1.538 (95% CI, 1.042 to 2.269), current-quit, 1.339 (95% CI, 1.077 to 1.666), and current-current, 1.589 (95% CI, 1.355 to 1.864).

Table 3 shows the HRs (95% CIs) for the incidence of gastric cancer according to smoking amount (pack-years). In the unadjusted model, the HRs (95% CIs) for the incidence of gastric cancer comparing light smokers, moderate smokers, and heavy smokers versus never smokers were 0.936 (95% CI, 0.783 to 1.118), 1.027 (95% CI, 0.864 to 1.221), and 1.289 (95% CI, 1.111 to 1.496), respectively (p for trend < 0.001). This association remained statistically significant, even after further adjustments for covariates in the multivariate-adjusted model: never smokers (reference); light smokers, 1.191 (95% CI, 0.992 to 1.430); moderate smokers, 1.298 (95% CI, 1.085 to 1.551); and heavy smokers, 1.410 (95% CI, 1.210 to 1.644) (p for trend < 0.001).

## DISCUSSION

The aim of this study was to quantify the risk of gastric cancer in Korean men according to changes in smoking status over time. We found that the risk of gastric cancer was relatively higher in men currently smoking in 2009 regardless of previous smoking status, compared with the never-never group. Although group 3 (never-current) had a widely dispersed 95% CI (HR, 1.347; 95% CI, 0.983 to 1.846), the 3 currently smoking groups in 2009 (never-current, quit-current, and current-current) had higher HRs for gastric cancer than the never-never group. This result suggested that a currently smoking status was the strongest risk factor for the future development of gastric cancer among all smoking status groups.

Our results are in line with previous studies that investigated the relationship between smoking and gastric cancer. Two meta-analyses of 46 case-control studies and 32 cohort studies demonstrated that “currently smoking” was associated with an increase in the risk of gastric cancer by 57% and 62%, respectively [12,13]. In a recent meta-analysis of 23 epidemiological works, current daily cigarette smoking showed an up to 32% elevation in the risk of gastric cancer [11]. These results are evidence that current smoking is causally linked to the development of gastric cancer. However, it is unclear whether former smoking is associated with an increased risk of gastric cancer. Our analysis showed that the former smoker groups (never-quit and quit-quit) did not have significantly higher HRs for incident gastric cancer compared with the never-never group. Previous studies have also demonstrated that the risk of gastric cancer in former smokers is lower than in current smokers, decreasing to the level of never smokers as the duration of smoking cessation increases [5-9]. Overall, our findings conclude that never smoking is optimal for preventing gastric cancer and that cessation of smoking is critical for current smokers.

The mechanisms by which smoking is involved in gastric carcinogenesis may help explain our results. Cigarettes contain multiple components (e.g., nicotine, aromatic amines, phenolic compounds, and N-nitroso compounds) that can be toxic and carcinogenic [14,15]. Studies have demonstrated that nicotine promotes gastric tumor growth and neovascularization [16]. Nicotine activates the nicotinic acetylcholine receptors on cancer cells and induces the release of growth factors such as vascular endothelial growth factor and interleukin-1β into the tumor microenvironment, which increases tumor angiogenesis and promotes tumor growth [17-19]. In a study of gastric cancer cases, levels of stable DNA adducts were significantly higher in the DNA of smokers than in the DNA of non-smokers [20]. N-nitroso compounds are carcinogens in tobacco that have been linked to the etiology of gastric cancer [21]. Additionally, smoking contributes to sequential changes from the gastric mucosa to precancerous lesions and then to cancer. A population-based gastroscopic screening study showed that smoking was significantly associated with precancerous lesions, including chronic atrophic gastritis and intestinal metaplasia and dysplasia [22]. The adverse effects of smoking may be most active in current smokers and less active in former smokers, depending on the duration of smoking cessation.

We assessed the risk of gastric cancer by smoking amount. Compared with never smokers, moderate smokers and heavy smokers showed an increased risk of gastric cancer in a dose-dependent pattern. This finding indicates that greater smoking amounts lead to a higher risk of gastric cancer. Interestingly, the former smokers in group 6 (current-quit) showed a significant association with an increased risk of gastric cancer. A plausible explanation for this finding may be the smoking amount in group 6. The mean smoking amount in group 6 was 23.2± 16.7 pack-years (heavy smokers). This finding suggests that high smoking amounts are linked to an increased risk of gastric cancer even in former smokers. Although never smoking is most effective at preventing gastric cancer, it is necessary to encourage smokers who are not ready to quit smoking to reduce their smoking amount.

The merits of the present study include reliable nationwide data, including serial evaluations for smoking status and diagnosis of gastric cancer based on ICD-10 codes. These advantages enabled us to quantify the risk of gastric cancer according to changes in smoking status and smoking amount.

Nonetheless, our study had several limitations. First, we could not identify *Helicobacter pylori* infection in our study participants because our data source did not contain information about *H. pylori* infection, which is a strong risk factor for gastric cancer. It has been reported that the adverse effect of *H. pylori* infection on gastric cancer can be exacerbated by smoking [23,24]. Butt et al. [6] demonstrated that an increased risk of gastric cancer in current smokers was only observed in individuals who were seropositive for *H. pylori*. Moreover, the prevalence of *H. pylori* infection is > 50% in Korean adults [25]. Therefore, the absence of data on *H. pylori* should be recognized as a major limitation in our study.

Second, the assessment of smoking status and amount was dependent on a self-reported questionnaire. Although the validity and reliability of the NHID have been confirmed in studies, recall bias is possible.

Third, there were no data for the location or histology of the gastric cancers in our study. Studies have shown heterogeneous results about whether smoking is primarily associated with cardia gastric cancer or with non-cardia gastric cancer [6-10]. However, information on the location of the gastric cancers in our data was not available.

In conclusion, current smoking was associated with an increased risk of gastric cancer, regardless of previous smoking status. Although former smoking was not associated with an increased risk of gastric cancer, former smokers with a history of heavy smoking had an increased risk of gastric cancer. These results suggest that smoking cessation and reducing smoking amounts are both important factors in reducing the risk of gastric cancer.

## Figures and Tables

**Figure f1-epih-44-e2022086:**
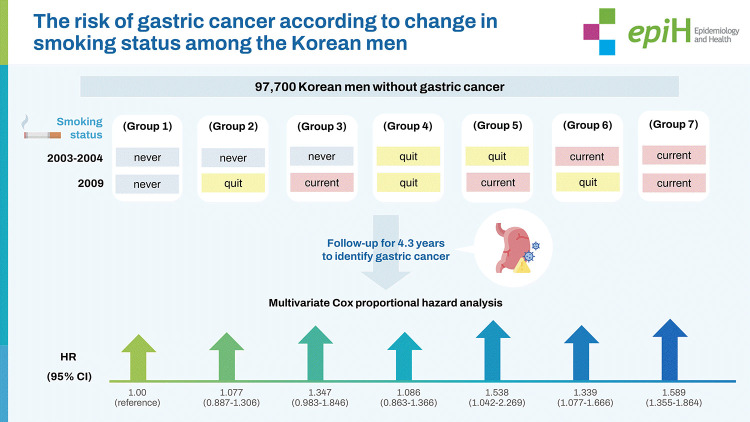


**Table 1.** Baseline characteristics of participants according to changes in smoking status (n=97,700)

Characteristics		Overall	Smoking status changes (from 2003-2004 to 2009)								p-value^1^
			2003-2004	Never (n=46,996, 48.10%)			Quit (n=12,849, 13.15%)		Current (n=37,855, 38.75%)		
			2009	Never group 1	Quit group 2	Current group 3	Quit group 4	Current group 5	Quit group 6	Current group 7	
Total (n)				29,117	13,863	4,016	10,525	2,324	10,226	27,629	
Proportion (%)				29.80	14.19	4.11	10.77	2.38	10.47	28.28	
Person-years (total)		423,288.7		126,629.6	60,057.6	17,300.8	45,734.4	10,018.9	44,357.5	119,189.9	
Person-years (average)		4.33±0.53		4.35±0.53	4.33±0.55	4.31±0.58	4.35±0.47	4.31±0.55	4.34±0.51	4.31±0.55	<0.001
Age (yr)		57.2±8.5		59.0±9.2	58.9±9.0	56.2±8.1	56.2±7.9	55.2±7.7	56.0±7.6	55.6±7.6	<0.001
BMI (kg/m^2^)		24.0±2.7		24.1±2.8	24.3±2.7	23.7±2.8	24.3±2.6	24.0±2.8	24.4±2.7	23.6±2.8	<0.001
Systolic BP (mmHg)		126.3±14.6		127.0±14.8	127.0±14.3	124.5±14.5	126.3±14.0	124.8±14.5	126.7±14.3	125.4±14.8	<0.001
Diastolic BP (mmHg)		78.8±9.7		79.0±9.9	78.9±9.6	78.1±9.7	79.0±9.5	78.5±9.7	79.3±9.6	78.5±9.8	<0.001
Fasting blood glucose (mg/dL)		102.9±27.0		102.0±25.7	102.9±25.0	102.9±28.9	102.8±24.2	102.9±28.2	104.9±28.4	103.2±29.3	<0.001
Total cholesterol (mg/dL)		195.9±36.5		193.3±35.4	194.8±36.4	197.1±36.7	196.7±35.5	197.4±38.5	199.0±37.3	197.7±37.1	<0.001
Triglyceride (mg/dL)		126 (88-184)		114 (80-166)	121 (85-175)	135 (94-199)	122 (86-177)	133 (92-197)	134 (93-196)	139 (96-202)	<0.001
HDL cholesterol (mg/dL)		52.9±28.3		53.5±30.9	53.0±27.5	52.4±30.4	53.1±23.7	51.4±22.0	53.0±25.9	52.4±28.3	<0.001
LDL cholesterol (mg/dL)		114.2±37.8		114.1±36.3	114.0±37.3	113.8±38.2	115.3±33.1	114.4±35.2	115.4±40.7	113.3±40.1	<0.001
AST (U/L)		25 (21-30)		25 (21-30)	25 (21-30)	24 (20-30)	25 (21-30)	24 (20-30)	25 (21-31)	24 (20-30)	<0.001
ALT (U/L)		23 (18-32)		23 (18-31)	23 (18-32)	23 (17-32)	23 (18-32)	23 (17-32)	24 (18-34)	23 (17-32)	<0.001
GGT (U/L)		33 (22-54)		28 (20-45)	30 (21-49)	35 (23-58)	32 (22-50)	36 (24-60)	37 (24-61)	38 (25-65)	<0.001
SCr (mg/dL)		1.00 (0.90-1.10)		1.00 (0.89-1.09)	1.00 (0.90-1.20)	1.00 (0.90-1.10)	1.00 (0.90-1.20)	1.00 (0.90-1.10)	1.00 (0.90-1.10)	1.00 (0.90-1.10)	<0.001
eGFR (mL/min per 1.73 m^2^)		79.9±21.2		79.3±20.3	78.3±20.8	81.9±19.8	77.8±23.9	80.3±22.9	79.5±21.8	81.9±20.9	<0.001
Smoking amount (pack years)^2^		15.0±16.2		0.0±0.0	15.7±14.8	21.6±15.2	17.4±14.1	19.8±14.3	23.2±16.7	25.2±14.7	<0.001
	Never smoker	29,117 (29.8)		(100)	0 (0.00)	0 (0.00)	0 (0.0)	0 (0.0)	0 (0.0)	0 (0.0)	<0.001
	Light smoker	20,016 (20.5)		0 (0.0)	6,834 (49.3)	1,111 (27.7)	4,293 (40.8)	753 (32.4)	2,611 (25.5)	4,414 (16.0)	
	Moderate smoker	20,046 (20.5)		0 (0.0)	3,837 (27.7)	1,122 (27.9)	3,406 (32.4)	641 (27.6)	2,987 (29.2)	8,053 (29.1)	
	Heavy smoker	28,521 (29.2)		0 (0.0)	3,192 (23.0)	1,783 (44.4)	2,826 (26.8)	930 (40.0)	4,628 (45.3)	15,162 (54.9)	
Alcohol intake		23,354 (24.1)		4,297 (14.9)	3,148 (22.9)	1,152 (28.9)	2,630 (25.2)	692 (30.0)	2,700 (26.6)	8,735 (31.8)	<0.001
Physical activity		17,713 (18.5)		5,587 (19.5)	3,074 (22.5)	593 (15.0)	2,234 (21.7)	360 (15.8)	2,007 (20.1)	3,858 (14.2)	<0.001
Incident gastric cancer		1,119 (1.1)		311 (1.1)	159 (1.1)	49 (1.2)	100 (0.9)	29 (1.2)	115 (1.1)	356 (1.3)	0.036

Values are presented as mean±standard deviation, medians (interquartile range), or number (%). BMI, body mass index; BP, blood pressure; HDL, high-density lipoprotein; LDL, low-density lipoprotein; AST, aspartate aminotransferase; ALT, alanine aminotransferase; GGT, gamma-glutamyl transferase; SCr, serum creatinine; eGFR, estimated glomerular filtration rate.

1 By the analysis of variance test for continuous variables and the chi-square test for categorical variables.

2 Light smoker: 0-10 pack years, moderate smoker: >10 pack years and ≤20 pack years, heavy smoker: >20 pack years.

**Table 2.** Hazard ratios (HRs) and 95% confidence intervals (CI) for incident gastric cancer according to changes in smoking status

Group	2003-2004	2009	Person-yr	Incident cases	Incidence density (per 10,000 person-yr)	HR (95% CI)	
						Unadjusted	Multivariate adjusted^1^
Group 1	Never	Never	126,629.6	311	24.5	1.000 (reference)	1.000 (reference)
Group 2	Never	Quit	60,057.6	159	26.5	1.078 (0.891, 1.305)	1.077 (0.887. 1.306)
Group 3	Never	Current	17,300.8	49	28.3	1.153 (0.853, 1.558)	1.347 (0.983, 1.846)
Group 4	Quit	Quit	45,734.4	100	21.9	0.891 (0.711, 1.116)	1.086 (0.863, 1.366)
Group 5	Quit	Current	10,018.9	29	28.9	1.179 (0.806, 1.725)	1.538 (1.042, 2.269)
Group 6	Current	Quit	44,357.5	115	25.9	1.056 (0.853, 1.308)	1.339 (1.077, 1.666)
Group 7	Current	Current	119,189.9	356	29.9	1.217 (1.045, 1.416)	1.589 (1.355, 1.864)

1 The multivariate-adjusted model was adjusted for age, body mass index, systolic blood pressure, fasting blood glucose level, total cholesterol level, alcohol intake, and physical activity.

**Table 3.** Hazard ratios (HRs) and 95% confidence intervals (CIs) for the incidence of gastric cancer according to 4 baseline levels of smoking amount in 2009

Variables	Person-yr	Incident cases	Incidence density (per 10,000 person-yr)	HR (95% CI)	
				Unadjusted	Multivariate adjusted^1^
Smoking amount (pack years)					
Never smoker	126,629.6	311	24.5	1.000 (reference)	1.000 (reference)
Light smoker (0-10)	86,668.4	199	23.0	0.936 (0.783, 1.118)	1.191 (0.992, 1.430)
Moderate smoker (10-20)	86,842.9	219	25.2	1.027 (0.864, 1.221)	1.298 (1.085, 1.551)
Heavy smoker (>20)	123,147.8	390	31.7	1.289 (1.111, 1.496)	1.410 (1.210, 1.644)
p for trend				<0.001	<0.001

1 The multivariate-adjusted model was adjusted for age, body mass index, systolic blood pressure, fasting blood glucose level, total cholesterol level, alcohol intake, and physical activity.
